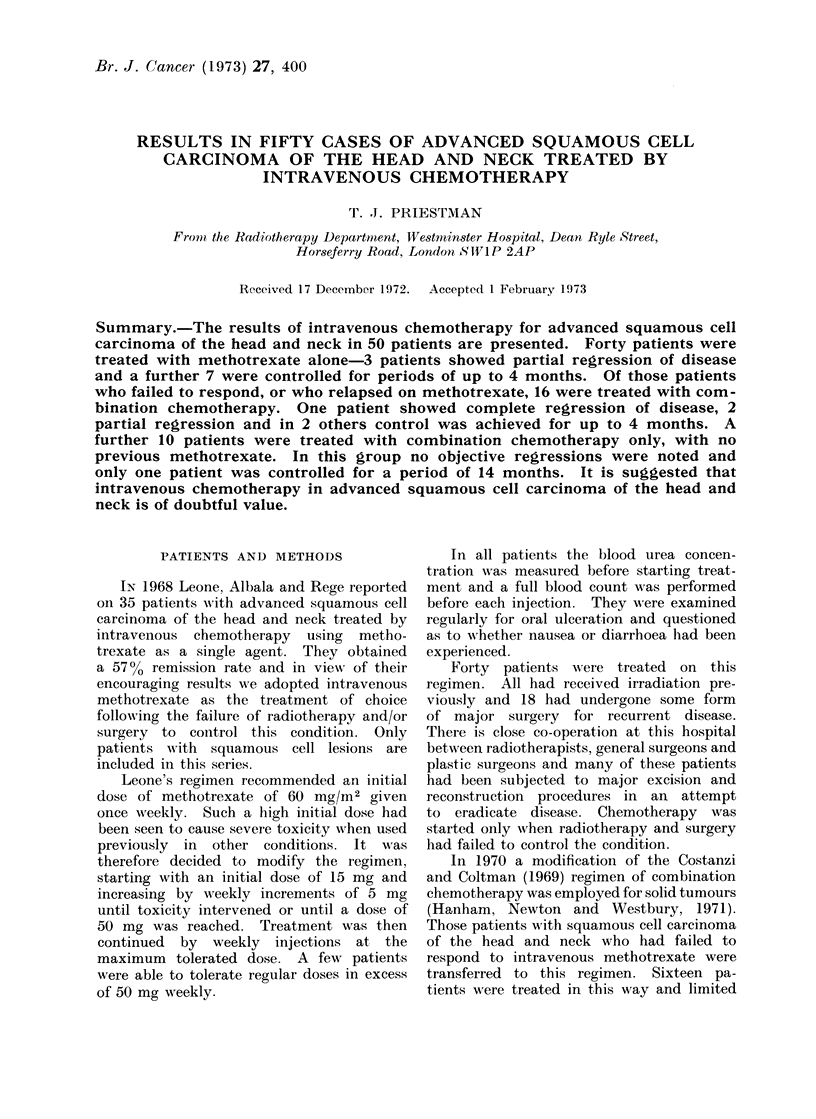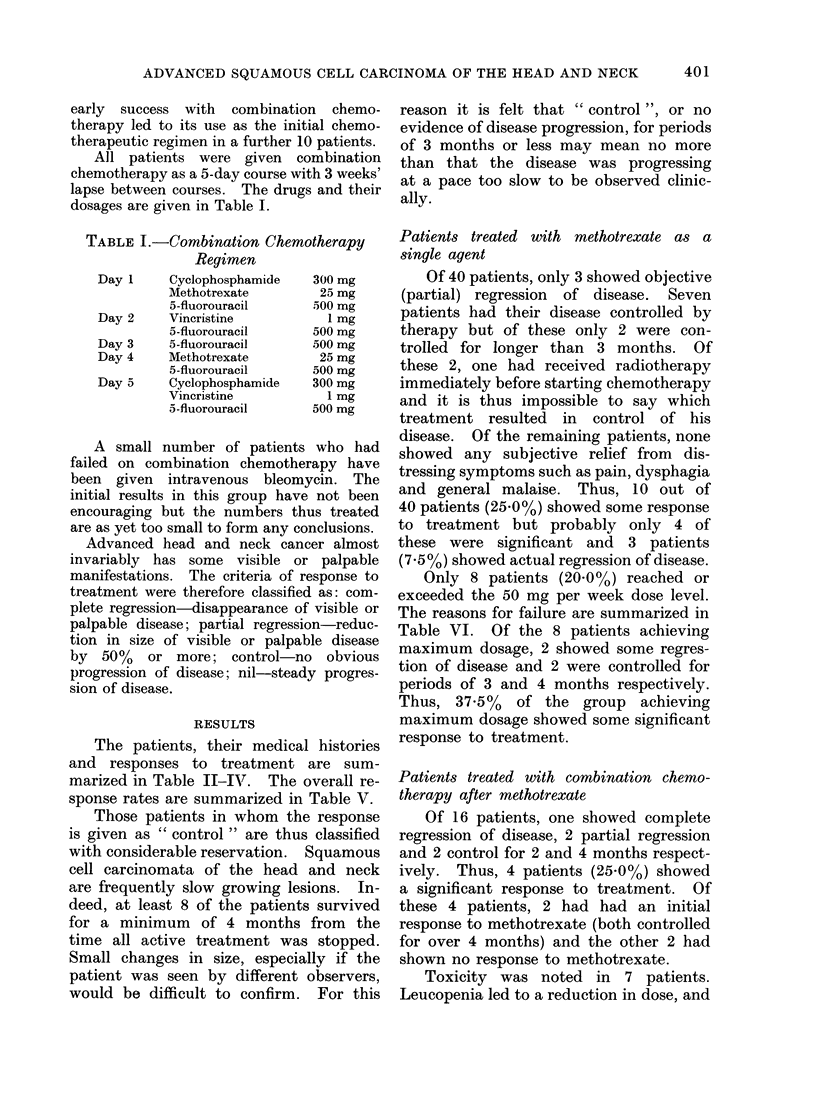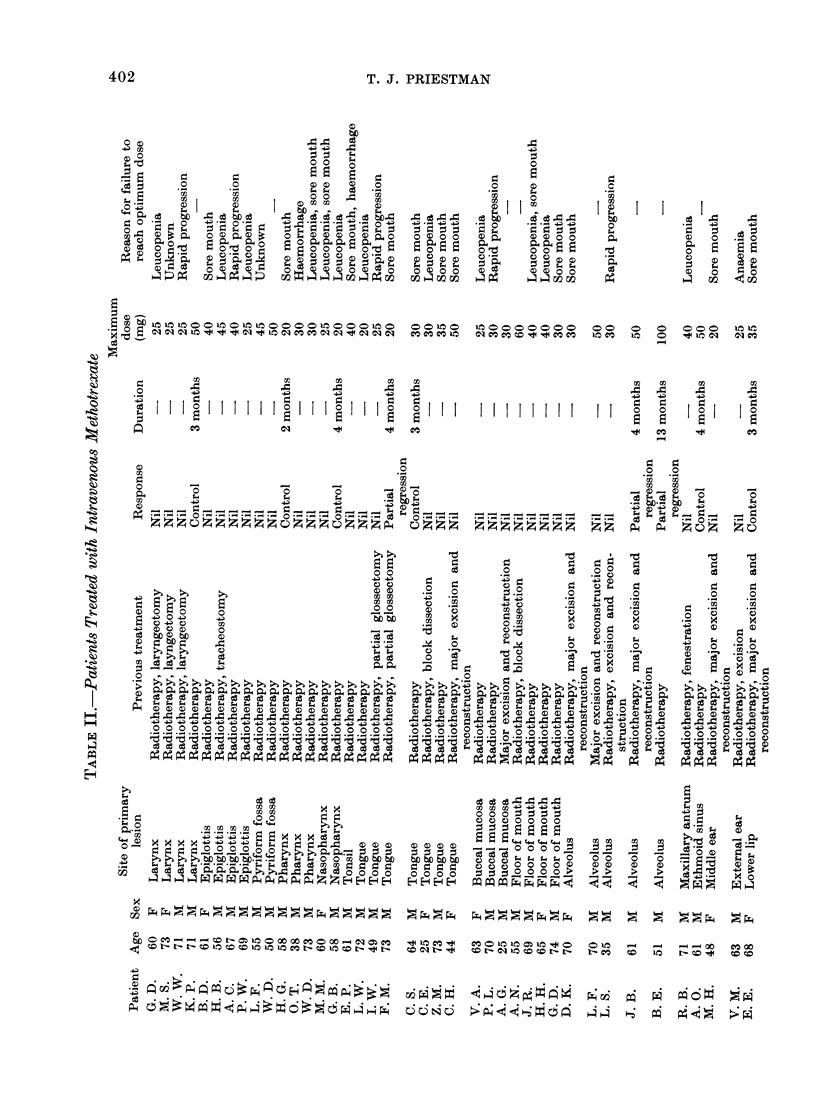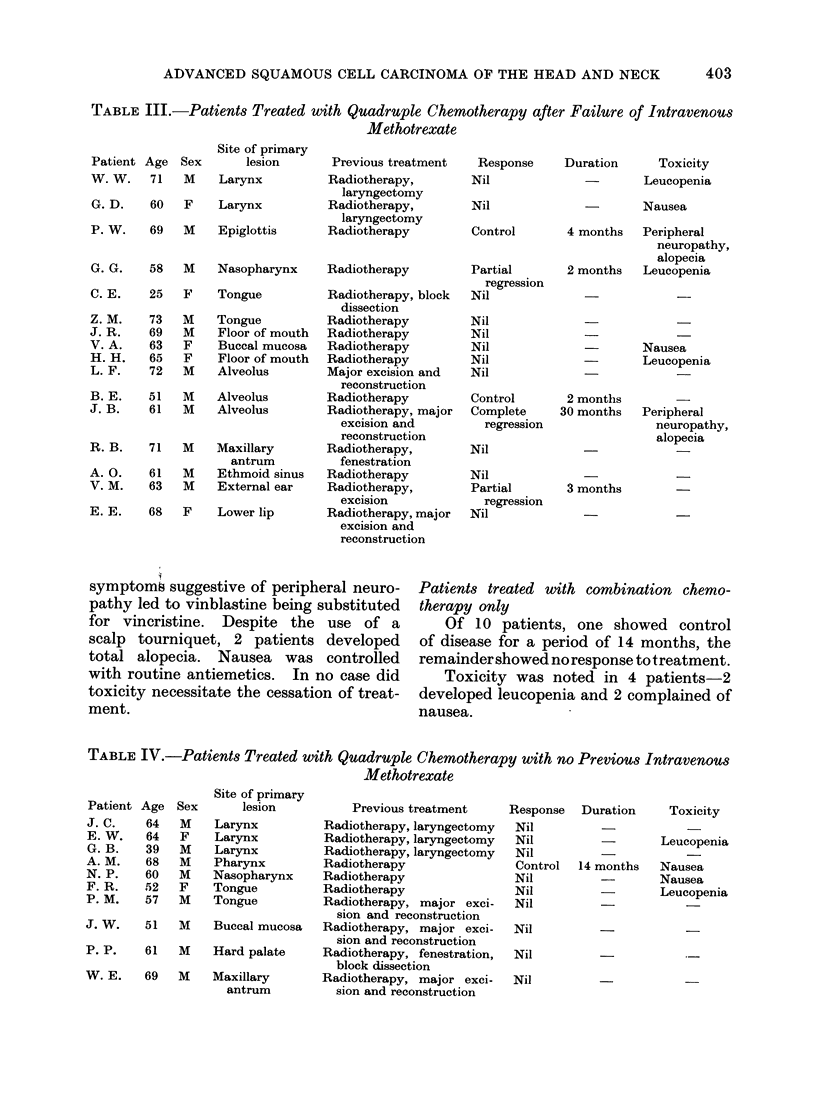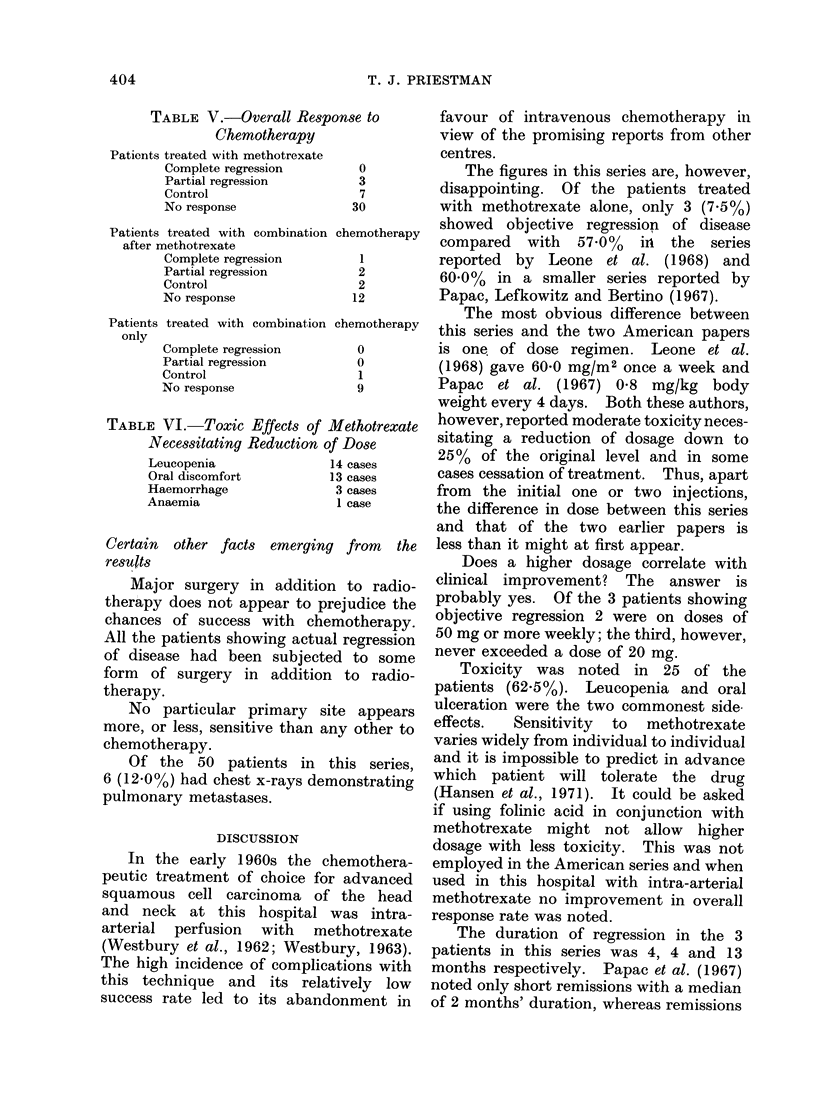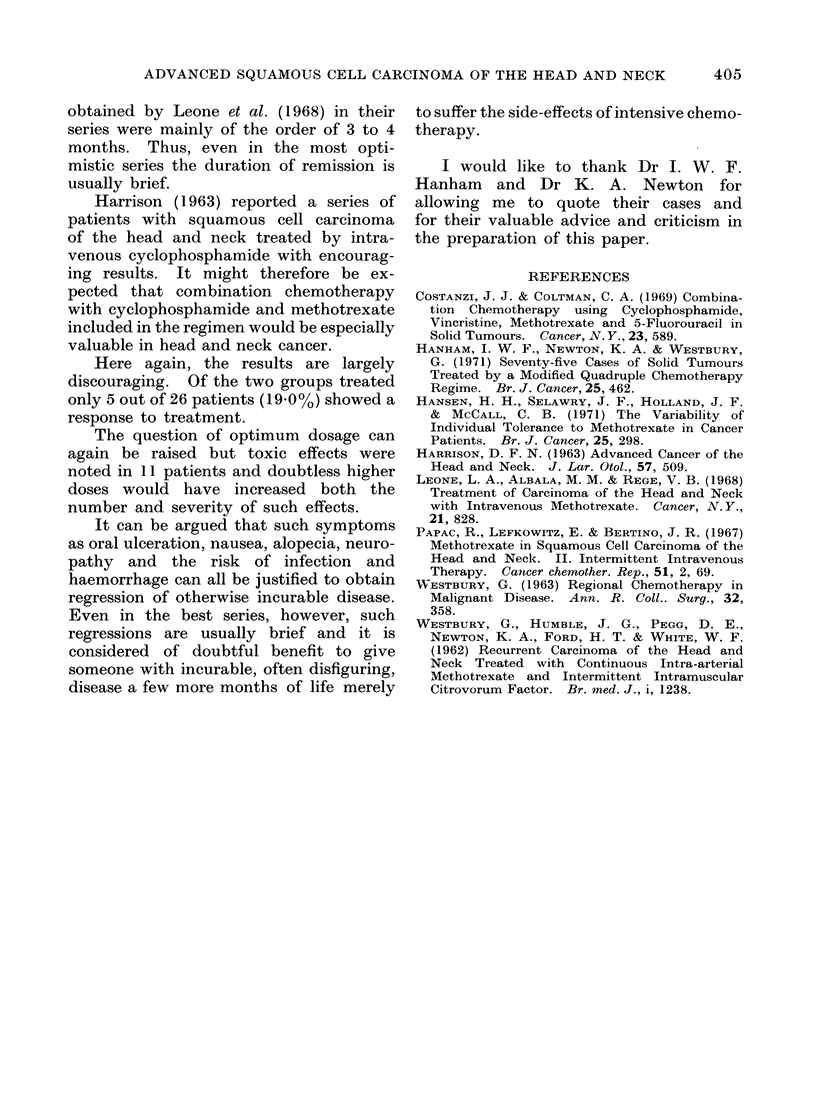# Results in Fifty Cases of Advanced Squamous Cell Carcinoma of the Head and Neck Treated by Intravenous Chemotherapy

**DOI:** 10.1038/bjc.1973.48

**Published:** 1973-05

**Authors:** T. J. Priestman

## Abstract

The results of intravenous chemotherapy for advanced squamous cell carcinoma of the head and neck in 50 patients are presented. Forty patients were treated with methotrexate alone—3 patients showed partial regression of disease and a further 7 were controlled for periods of up to 4 months. Of those patients who failed to respond, or who relapsed on methotrexate, 16 were treated with combination chemotherapy. One patient showed complete regression of disease, 2 partial regression and in 2 others control was achieved for up to 4 months. A further 10 patients were treated with combination chemotherapy only, with no previous methotrexate. In this group no objective regressions were noted and only one patient was controlled for a period of 14 months. It is suggested that intravenous chemotherapy in advanced squamous cell carcinoma of the head and neck is of doubtful value.


Br. J. Cancer (1973) 27, 400

### RESULTS IN FIFTY CASES OF ADVANCED SQUAMOUS CELL CARCINOMA OF THE HEAD AND NECK TREATED BY INTRAVENOUS CHEMOTHERAPY

T. J. PRIESTMAN

*From the Radiotherapy Department, Westminster Hospital, Dean Ryle Street, Horseferry Road, London SW1P 2AP*

Received 17 December 1972. Accepted 1 February 1973

**Summary.—The results of intravenous chemotherapy for advanced squamous cell carcinoma of the head and neck in 50 patients are presented. Forty patients were treated with methotrexate alone—3 patients showed partial regression of disease and a further 7 were controlled for periods of up to 4 months. Of those patients who failed to respond, or who relapsed on methotrexate, 16 were treated with combination chemotherapy. One patient showed complete regression of disease, 2 partial regression and in 2 others control was achieved for up to 4 months. A further 10 patients were treated with combination chemotherapy only, with no previous methotrexate. In this group no objective regressions were noted and only one patient was controlled for a period of 14 months. It is suggested that intravenous chemotherapy in advanced squamous cell carcinoma of the head and neck is of doubtful value.**

### PATIENTS AND METHODS

In 1968 Leone, Albala and Rege reported on 35 patients with advanced squamous cell carcinoma of the head and neck treated by intravenous chemotherapy using methotrexate as a single agent. They obtained a 57% remission rate and in view of their encouraging results we adopted intravenous methotrexate as the treatment of choice following the failure of radiotherapy and/or surgery to control this condition. Only patients with squamous cell lesions are included in this series.

Leone's regimen recommended an initial dose of methotrexate of 60 mg/m$^2$ given once weekly. Such a high initial dose had been seen to cause severe toxicity when used previously in other conditions. It was therefore decided to modify the regimen, starting with an initial dose of 15 mg and increasing by weekly increments of 5 mg until toxicity intervened or until a dose of 50 mg was reached. Treatment was then continued by weekly injections at the maximum tolerated dose. A few patients were able to tolerate regular doses in excess of 50 mg weekly.

In all patients the blood urea concentration was measured before starting treatment and a full blood count was performed before each injection. They were examined regularly for oral ulceration and questioned as to whether nausea or diarrhoea had been experienced.

Forty patients were treated on this regimen. All had received irradiation previously and 18 had undergone some form of major surgery for recurrent disease. There is close co-operation at this hospital between radiotherapists, general surgeons and plastic surgeons and many of these patients had been subjected to major excision and reconstruction procedures in an attempt to eradicate disease. Chemotherapy was started only when radiotherapy and surgery had failed to control the condition.

In 1970 a modification of the Costanzi and Coltman (1969) regimen of combination chemotherapy was employed for solid tumours (Hanham, Newton and Westbury, 1971). Those patients with squamous cell carcinoma of the head and neck who had failed to respond to intravenous methotrexate were transferred to this regimen. Sixteen patients were treated in this way and limited early success with combination chemotherapy led to its use as the initial chemotherapeutic regimen in a further 10 patients.

All patients were given combination chemotherapy as a 5-day course with 3 weeks' lapse between courses. The drugs and their dosages are given in Table I.

TABLE I.—*Combination Chemotherapy Regimen*

| | | |
|---|---|---|
| Day 1 | Cyclophosphamide | 300 mg |
| | Methotrexate | 25 mg |
| | 5-fluorouracil | 500 mg |
| Day 2 | Vincristine | 1 mg |
| | 5-fluorouracil | 500 mg |
| Day 3 | 5-fluorouracil | 500 mg |
| Day 4 | Methotrexate | 25 mg |
| | 5-fluorouracil | 500 mg |
| Day 5 | Cyclophosphamide | 300 mg |
| | Vincristine | 1 mg |
| | 5-fluorouracil | 500 mg |

A small number of patients who had failed on combination chemotherapy have been given intravenous bleomycin. The initial results in this group have not been encouraging but the numbers thus treated are as yet too small to form any conclusions.

Advanced head and neck cancer almost invariably has some visible or palpable manifestations. The criteria of response to treatment were therefore classified as: complete regression—disappearance of visible or palpable disease; partial regression—reduction in size of visible or palpable disease by 50% or more; control—no obvious progression of disease; nil—steady progression of disease.

### RESULTS

The patients, their medical histories and responses to treatment are summarized in Table II–IV. The overall response rates are summarized in Table V.

Those patients in whom the response is given as " control " are thus classified with considerable reservation. Squamous cell carcinomata of the head and neck are frequently slow growing lesions. Indeed, at least 8 of the patients survived for a minimum of 4 months from the time all active treatment was stopped. Small changes in size, especially if the patient was seen by different observers, would be difficult to confirm. For this reason it is felt that " control ", or no evidence of disease progression, for periods of 3 months or less may mean no more than that the disease was progressing at a pace too slow to be observed clinically.

*Patients treated with methotrexate as a single agent*

Of 40 patients, only 3 showed objective (partial) regression of disease. Seven patients had their disease controlled by therapy but of these only 2 were controlled for longer than 3 months. Of these 2, one had received radiotherapy immediately before starting chemotherapy and it is thus impossible to say which treatment resulted in control of his disease. Of the remaining patients, none showed any subjective relief from distressing symptoms such as pain, dysphagia and general malaise. Thus, 10 out of 40 patients (25·0%) showed some response to treatment but probably only 4 of these were significant and 3 patients (7·5%) showed actual regression of disease.

Only 8 patients (20·0%) reached or exceeded the 50 mg per week dose level. The reasons for failure are summarized in Table VI. Of the 8 patients achieving maximum dosage, 2 showed some regression of disease and 2 were controlled for periods of 3 and 4 months respectively. Thus, 37·5% of the group achieving maximum dosage showed some significant response to treatment.

*Patients treated with combination chemotherapy after methotrexate*

Of 16 patients, one showed complete regression of disease, 2 partial regression and 2 control for 2 and 4 months respectively. Thus, 4 patients (25·0%) showed a significant response to treatment. Of these 4 patients, 2 had had an initial response to methotrexate (both controlled for over 4 months) and the other 2 had shown no response to methotrexate.

Toxicity was noted in 7 patients. Leucopenia led to a reduction in dose, and

TABLE II.—*Patients Treated with Intravenous Methotrexate*

| Patient | Age | Sex | Site of primary lesion | Previous treatment | Response | Duration | Maximum dose (mg) | Reason for failure to reach optimum dose |
|---|---|---|---|---|---|---|---|---|
| G. D. | 60 | F | Larynx | Radiotherapy, laryngectomy | Nil | — | 25 | Leucopenia |
| M. S. | 73 | F | Larynx | Radiotherapy, layngectomy | Nil | — | 25 | Unknown |
| W. W. | 71 | M | Larynx | Radiotherapy, laryngectomy | Nil | — | 25 | Rapid progression |
| K. P. | 71 | M | Larynx | Radiotherapy | Control | 3 months | 50 | — |
| B. D. | 61 | F | Epiglottis | Radiotherapy | Nil | — | 40 | Sore mouth |
| H. B. | 56 | M | Epiglottis | Radiotherapy, tracheostomy | Nil | — | 45 | Leucopenia |
| A. C. | 67 | M | Epiglottis | Radiotherapy | Nil | — | 40 | Rapid progression |
| P. W. | 69 | M | Epiglottis | Radiotherapy | Nil | — | 25 | Leucopenia |
| L. F. | 55 | M | Pyriform fossa | Radiotherapy | Nil | — | 45 | Unknown |
| W. D. | 50 | M | Pyriform fossa | Radiotherapy | Nil | — | 50 | — |
| H. G. | 58 | M | Pharynx | Radiotherapy | Control | 2 months | 20 | Sore mouth |
| O. T. | 38 | M | Pharynx | Radiotherapy | Nil | — | 30 | Haemorrhage |
| W. D. | 73 | M | Pharynx | Radiotherapy | Nil | — | 30 | Leucopenia, sore mouth |
| M. M. | 60 | F | Nasopharynx | Radiotherapy | Nil | — | 25 | Leucopenia, sore mouth |
| G. B. | 58 | M | Nasopharynx | Radiotherapy | Control | 4 months | 20 | Leucopenia |
| E. P. | 61 | M | Tonsil | Radiotherapy | Nil | — | 40 | Sore mouth, haemorrhage |
| L. W. | 72 | M | Tongue | Radiotherapy | Nil | — | 20 | Leucopenia |
| I. W. | 49 | M | Tongue | Radiotherapy, partial glossectomy | Nil | — | 25 | Rapid progression |
| F. M. | 73 | M | Tongue | Radiotherapy, partial glossectomy | Partial regression | 4 months | 20 | Sore mouth |
| C. S. | 64 | M | Tongue | Radiotherapy | Control | 3 months | 30 | Sore mouth |
| C. E. | 25 | F | Tongue | Radiotherapy, block dissection | Nil | — | 30 | Leucopenia |
| Z. M. | 73 | M | Tongue | Radiotherapy | Nil | — | 35 | Sore mouth |
| C. H. | 44 | F | Tongue | Radiotherapy, major excision and reconstruction | Nil | — | 50 | Sore mouth |
| V. A. | 63 | F | Buccal mucosa | Radiotherapy | Nil | — | 25 | Leucopenia |
| P. L. | 70 | M | Buccal mucosa | Radiotherapy | Nil | — | 30 | Rapid progression |
| A. G. | 25 | M | Buccal mucosa | Major excision and reconstruction | Nil | — | 30 | — |
| A. N. | 55 | M | Floor of mouth | Radiotherapy, block dissection | Nil | — | 60 | — |
| J. R. | 69 | M | Floor of mouth | Radiotherapy | Nil | — | 40 | Leucopenia, sore mouth |
| H. H. | 65 | F | Floor of mouth | Radiotherapy | Nil | — | 40 | Leucopenia |
| G. D. | 74 | M | Floor of mouth | Radiotherapy | Nil | — | 30 | Sore mouth |
| D. K. | 70 | F | Alveolus | Radiotherapy, major excision and reconstruction | Nil | — | 30 | Sore mouth |
| L. F. | 70 | M | Alveolus | Major excision and reconstruction | Nil | — | 50 | — |
| L. S. | 35 | M | Alveolus | Radiotherapy, excision and reconstruction | Nil | — | 30 | Rapid progression |
| J. B. | 61 | M | Alveolus | Radiotherapy, major excision and reconstruction | Partial regression | 4 months | 50 | — |
| B. E. | 51 | M | Alveolus | Radiotherapy | Partial regression | 13 months | 100 | — |
| R. B. | 71 | M | Maxillary antrum | Radiotherapy, fenestration | Nil | — | 40 | Leucopenia |
| A. O. | 61 | M | Ethmoid sinus | Radiotherapy | Control | 4 months | 50 | — |
| M. H. | 48 | F | Middle ear | Radiotherapy, major excision and reconstruction | Nil | — | 20 | Sore mouth |
| V. M. | 63 | M | External ear | Radiotherapy, excision | Nil | — | 25 | Anaemia |
| E. E. | 68 | F | Lower lip | Radiotherapy, major excision and reconstruction | Control | 3 months | 35 | Sore mouth |

TABLE III.—*Patients Treated with Quadruple Chemotherapy after Failure of Intravenous Methotrexate*

| Patient | Age | Sex | Site of primary lesion | Previous treatment | Response | Duration | Toxicity |
|---|---|---|---|---|---|---|---|
| W. W. | 71 | M | Larynx | Radiotherapy, laryngectomy | Nil | — | Leucopenia |
| G. D. | 60 | F | Larynx | Radiotherapy, laryngectomy | Nil | — | Nausea |
| P. W. | 69 | M | Epiglottis | Radiotherapy | Control | 4 months | Peripheral neuropathy, alopecia |
| G. G. | 58 | M | Nasopharynx | Radiotherapy | Partial regression | 2 months | Leucopenia |
| C. E. | 25 | F | Tongue | Radiotherapy, block dissection | Nil | — | — |
| Z. M. | 73 | M | Tongue | Radiotherapy | Nil | — | — |
| J. R. | 69 | M | Floor of mouth | Radiotherapy | Nil | — | — |
| V. A. | 63 | F | Buccal mucosa | Radiotherapy | Nil | — | Nausea |
| H. H. | 65 | F | Floor of mouth | Radiotherapy | Nil | — | Leucopenia |
| L. F. | 72 | M | Alveolus | Major excision and reconstruction | Nil | — | — |
| B. E. | 51 | M | Alveolus | Radiotherapy | Control | 2 months | — |
| J. B. | 61 | M | Alveolus | Radiotherapy, major excision and reconstruction | Complete regression | 30 months | Peripheral neuropathy, alopecia |
| R. B. | 71 | M | Maxillary antrum | Radiotherapy, fenestration | Nil | — | — |
| A. O. | 61 | M | Ethmoid sinus | Radiotherapy | Nil | — | — |
| V. M. | 63 | M | External ear | Radiotherapy, excision | Partial regression | 3 months | — |
| E. E. | 68 | F | Lower lip | Radiotherapy, major excision and reconstruction | Nil | — | — |

symptoms suggestive of peripheral neuropathy led to vinblastine being substituted for vincristine. Despite the use of a scalp tourniquet, 2 patients developed total alopecia. Nausea was controlled with routine antiemetics. In no case did toxicity necessitate the cessation of treatment.

*Patients treated with combination chemotherapy only*

Of 10 patients, one showed control of disease for a period of 14 months, the remainder showed no response to treatment.

Toxicity was noted in 4 patients—2 developed leucopenia and 2 complained of nausea.

TABLE IV.—*Patients Treated with Quadruple Chemotherapy with no Previous Intravenous Methotrexate*

| Patient | Age | Sex | Site of primary lesion | Previous treatment | Response | Duration | Toxicity |
|---|---|---|---|---|---|---|---|
| J. C. | 64 | M | Larynx | Radiotherapy, laryngectomy | Nil | — | — |
| E. W. | 64 | F | Larynx | Radiotherapy, laryngectomy | Nil | — | Leucopenia |
| G. B. | 39 | M | Larynx | Radiotherapy, laryngectomy | Nil | — | — |
| A. M. | 68 | M | Pharynx | Radiotherapy | Control | 14 months | Nausea |
| N. P. | 60 | M | Nasopharynx | Radiotherapy | Nil | — | Nausea |
| F. R. | 52 | F | Tongue | Radiotherapy | Nil | — | Leucopenia |
| P. M. | 57 | M | Tongue | Radiotherapy, major excision and reconstruction | Nil | — | — |
| J. W. | 51 | M | Buccal mucosa | Radiotherapy, major excision and reconstruction | Nil | — | — |
| P. P. | 61 | M | Hard palate | Radiotherapy, fenestration, block dissection | Nil | — | — |
| W. E. | 69 | M | Maxillary antrum | Radiotherapy, major excision and reconstruction | Nil | — | — |

TABLE V.—*Overall Response to Chemotherapy*

| | |
|---|---|
| Patients treated with methotrexate | |
| Complete regression | 0 |
| Partial regression | 3 |
| Control | 7 |
| No response | 30 |
| Patients treated with combination chemotherapy after methotrexate | |
| Complete regression | 1 |
| Partial regression | 2 |
| Control | 2 |
| No response | 12 |
| Patients treated with combination chemotherapy only | |
| Complete regression | 0 |
| Partial regression | 0 |
| Control | 1 |
| No response | 9 |

TABLE VI.—*Toxic Effects of Methotrexate Necessitating Reduction of Dose*

| | |
|---|---|
| Leucopenia | 14 cases |
| Oral discomfort | 13 cases |
| Haemorrhage | 3 cases |
| Anaemia | 1 case |

*Certain other facts emerging from the results*

Major surgery in addition to radiotherapy does not appear to prejudice the chances of success with chemotherapy. All the patients showing actual regression of disease had been subjected to some form of surgery in addition to radiotherapy.

No particular primary site appears more, or less, sensitive than any other to chemotherapy.

Of the 50 patients in this series, 6 (12·0%) had chest x-rays demonstrating pulmonary metastases.

### DISCUSSION

In the early 1960s the chemotherapeutic treatment of choice for advanced squamous cell carcinoma of the head and neck at this hospital was intra-arterial perfusion with methotrexate (Westbury *et al.*, 1962; Westbury, 1963). The high incidence of complications with this technique and its relatively low success rate led to its abandonment in favour of intravenous chemotherapy in view of the promising reports from other centres.

The figures in this series are, however, disappointing. Of the patients treated with methotrexate alone, only 3 (7·5%) showed objective regression of disease compared with 57·0% in the series reported by Leone *et al.* (1968) and 60·0% in a smaller series reported by Papac, Lefkowitz and Bertino (1967).

The most obvious difference between this series and the two American papers is one of dose regimen. Leone *et al.* (1968) gave 60·0 mg/m$^2$ once a week and Papac *et al.* (1967) 0·8 mg/kg body weight every 4 days. Both these authors, however, reported moderate toxicity necessitating a reduction of dosage down to 25% of the original level and in some cases cessation of treatment. Thus, apart from the initial one or two injections, the difference in dose between this series and that of the two earlier papers is less than it might at first appear.

Does a higher dosage correlate with clinical improvement? The answer is probably yes. Of the 3 patients showing objective regression 2 were on doses of 50 mg or more weekly; the third, however, never exceeded a dose of 20 mg.

Toxicity was noted in 25 of the patients (62·5%). Leucopenia and oral ulceration were the two commonest side-effects. Sensitivity to methotrexate varies widely from individual to individual and it is impossible to predict in advance which patient will tolerate the drug (Hansen *et al.*, 1971). It could be asked if using folinic acid in conjunction with methotrexate might not allow higher dosage with less toxicity. This was not employed in the American series and when used in this hospital with intra-arterial methotrexate no improvement in overall response rate was noted.

The duration of regression in the 3 patients in this series was 4, 4 and 13 months respectively. Papac *et al.* (1967) noted only short remissions with a median of 2 months' duration, whereas remissions obtained by Leone *et al.* (1968) in their series were mainly of the order of 3 to 4 months. Thus, even in the most optimistic series the duration of remission is usually brief.

Harrison (1963) reported a series of patients with squamous cell carcinoma of the head and neck treated by intravenous cyclophosphamide with encouraging results. It might therefore be expected that combination chemotherapy with cyclophosphamide and methotrexate included in the regimen would be especially valuable in head and neck cancer.

Here again, the results are largely discouraging. Of the two groups treated only 5 out of 26 patients (19·0%) showed a response to treatment.

The question of optimum dosage can again be raised but toxic effects were noted in 11 patients and doubtless higher doses would have increased both the number and severity of such effects.

It can be argued that such symptoms as oral ulceration, nausea, alopecia, neuropathy and the risk of infection and haemorrhage can all be justified to obtain regression of otherwise incurable disease. Even in the best series, however, such regressions are usually brief and it is considered of doubtful benefit to give someone with incurable, often disfiguring, disease a few more months of life merely to suffer the side-effects of intensive chemotherapy.

I would like to thank Dr I. W. F. Hanham and Dr K. A. Newton for allowing me to quote their cases and for their valuable advice and criticism in the preparation of this paper.

### REFERENCES

Costanzi, J. J. & Coltman, C. A. (1969) Combination Chemotherapy using Cyclophosphamide, Vincristine, Methotrexate and 5-Fluorouracil in Solid Tumours. *Cancer, N.Y.*, **23**, 589.

Hanham, I. W. F., Newton, K. A. & Westbury, G. (1971) Seventy-five Cases of Solid Tumours Treated by a Modified Quadruple Chemotherapy Regime. *Br. J. Cancer*, **25**, 462.

Hansen, H. H., Selawry, J. F., Holland, J. F. & McCall, C. B. (1971) The Variability of Individual Tolerance to Methotrexate in Cancer Patients. *Br. J. Cancer*, **25**, 298.

Harrison, D. F. N. (1963) Advanced Cancer of the Head and Neck. *J. Lar. Otol.*, **57**, 509.

Leone, L. A., Albala, M. M. & Rege, V. B. (1968) Treatment of Carcinoma of the Head and Neck with Intravenous Methotrexate. *Cancer, N.Y.*, **21**, 828.

Papac, R., Lefkowitz, E. & Bertino, J. R. (1967) Methotrexate in Squamous Cell Carcinoma of the Head and Neck. II. Intermittent Intravenous Therapy. *Cancer chemother. Rep.*, **51**, 2, 69.

Westbury, G. (1963) Regional Chemotherapy in Malignant Disease. *Ann. R. Coll.. Surg.*, **32**, 358.

Westbury, G., Humble, J. G., Pegg, D. E., Newton, K. A., Ford, H. T. & White, W. F. (1962) Recurrent Carcinoma of the Head and Neck Treated with Continuous Intra-arterial Methotrexate and Intermittent Intramuscular Citrovorum Factor. *Br. med. J.*, i, 1238.